# Increasing workflow development speed and reproducibility with Vectools

**DOI:** 10.12688/f1000research.16301.2

**Published:** 2018-10-23

**Authors:** Tyler Weirick, Raphael Müller, Shizuka Uchida

**Affiliations:** 1 Cardiovascular Innovation Institute, University of Louisville, Louisville, KY, 40202, USA; 2 Institute of Cardiovascular Regeneration, Goethe University Frankfurt, Frankfurt am Main, Hessen, 60590, Germany; 3 Institute for Bioinformatics and Systems Biology, Justus Liebig University Giessen, Giessen, Hessen, 35392, Germany; 4 Institute of Molecular Cardiology, University of Louisville, Louisville, KY, 40202, USA

**Keywords:** bioinformatics, reproducibility, workflow, vector, matrix, spreadsheet

## Abstract

Despite advances in bioinformatics, custom scripts remain a source of difficulty, slowing workflow development and hampering reproducibility. Here, we introduce Vectools, a command-line tool-suite to reduce reliance on custom scripts and improve reproducibility by offering a wide range of common easy-to-use functions for table and vector manipulation. Vectools also offers a number of vector related functions to speed up workflow development, such as simple machine learning and common statistics functions.

## Introduction

Although the importance of computational analyses in biological research is increasingly appreciated, many analyses are time consuming to implement and remain complicated, as well as being difficult to reproduce ^1^. Workflow-managers [e.g., Snakemake ^2^] have greatly simplified many aspects needed for reproducibility. However, custom scripts (i.e., software not intended for use by a wider audience) remain a problem, which hampers the increased shareability offered by workflow-managers ^3^. Custom scripts are often needed to further process data generated by high-use programs (i.e., programs intended for a wide user base). At the most basic level, analysis pipelines requiring custom scripts simply take more time to implement as additional code needs to be written. In addition, writing custom scripts also increases the chance of software bugs, which is concerning as even small bugs have led to retractions, such as mislabeling metadata ^4^ or a sign change ^5^. Furthermore, analyses using custom scripts also hamper reproducibility as the scripts may be publically unavailable, lack documentation, or does not work on certain operation systems. To reduce the impact of these problems, we introduce Vectools ^6^, a command-line tool for working with vectors, matrices, and tables. Vectools reduces the need for custom scripts by offering an easy-to-use command-line tool with a wide range functions for manipulating tables, one of the most commonly used formats in bioinformatics. Further, Vectools incorporates a number of other useful vector-related functions, such as statistics and machine learning. Altogether, Vectools helps to speed up workflow development and improves reproducibility by offering a wide range of useful functions.

## Methods

### Implementation

Vectools can be run via command-line by simply typing “vectools”, which will print the main help menu. Vectools contains over 45 operations organized by headings. These are analysis, descriptors, manipulation, math, normalization, supervised learning, and unsupervised learning. A list of all headings and functions is available in ( Supplementary File 1). To run an operation, simply type “vectools” followed by the operation name. If the “—help” argument is added after an operation name, a help menu with usage instructions and examples will be printed.

### Operation

A standard laptop computer with a recent version of Python3 will handle most applications.

## Use cases

**Figure 1.**
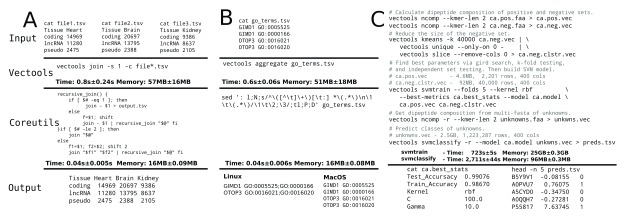
Comparison of Vectools and Coreutils. ( **A**) Joining more than two files requires a single command using Vectools. The same operation using Coreutils requires a custom script. The information regarding file sizes is omitted as whole files are shown. ( **B**) Aggregating Gene Ontology terms by gene accession numbers with Vectools can be done with a simple command. The same operation using Coreutils requires a complex regular expression. Further, the regular expression does not work properly on MacOS. The information regarding file sizes is omitted as whole files are shown. ( **C**) Vectools also includes many operations unavailable in Coreutils, such as machine learning. Here, in five commands, we use supervised-learning for homology-independent prediction of enzyme function. Using Vectools we generated a support-vector machine model capable of predicting carbonic anhydrases with an estimated 99% accuracy and predict 15,018 of 1,223,287 uncharacterized proteins as potential carbonic anhydrases. The size and dimensions of files used in the machine learning examples are shown in the image as comments. Additionally, methods, input, and output data can be found in the archived data and analysis pipelines ^7^.

When manipulating data in tables, Core Utilities (Coreutils) programs (e.g., awk, grep, sed, and join) can be used instead of custom scripts. Using Coreutils helps to solve problems with availability as they are common to Unix-based systems. Here, we compared the usage of Vectools to Coreutils. Methods and output can be found in the archived data ^7^. One downside of Coreutils programs is that they can be complex and difficult to understand. For example, joining multiple tables requires a Bash script using Coreutils-join, whereas this can be done with a single line with Vectools ( Figure 1A). Furthermore, while common in Unix systems, the behavior of Coreutils programs may differ depending on the operating system. These differences can potentially cause errors or unexpected behavior, such as aggregating Gene Ontology (GO) terms by gene accession numbers with sed ( Figure 1B). Instead of aggregating values on MacOS or other Berkeley Software Distribution (BSD) Unix systems, the Coreutils function prints the original input data. These errors can be caused by multiple reasons, such as BSD-sed not interpreting ANSI-C escape sequences (e.g., \n for newline, \t for tab) and differences in how regular expressions are evaluated. These problems can be overcome with Vectools with only one line of command. Vectools offers many functions that are currently unavailable in Coreutils, such as basic machine learning. Here, we show a simple example of using a support-vector machine to find potential novel carbonic anhydrases independent of sequence homology ( Figure 1C). Carbonic anhydrases were chosen as they have multiple distinct classes, which arose via convergent evolution ^8^. Vectools significantly simplifies a number of steps needed for this task. For example, the “svmtrain” operation handles hyper-parameter tuning via grid search, k-fold testing, and independent set testing. This significantly simplifies implementing machine learning in analysis pipelines.

## Discussion

Here, we show that Vectools reduces the need for custom scripts and is simpler to use than Coreutils. While Coreutils is faster and uses less memory, this is generally a minor issue given the increasing power and decreasing cost of computational resources. Although format-specific tools (e.g., Bedtools ^9^) offer similar functionalities, the generalized design of Vectools allows the majority of these functionalities to be replicated by combining Vectools operations with pipes. Furthermore, Vectools includes various other functionalities not available in Coreutils or format-specific tools, such as allowing easy incorporation of machine learning into analysis pipelines. Users may also be interested in comparison with R. While certainly suited to the same tasks: 1) integrating R into a pipeline requires custom scripts; and 2) the use-cases for R and Vectools are different. R offers a large variety of functions at the cost of package dependency issues. Conversely, Vectools emphasizes ease-of-use by hosting a curated list of common functions, helps to increase reproducibility by making analysis pipelines easier to share, and reduces bugs by omitting the need for custom scripts. Thus, one common use-case of Vectools when combined with a workflow-manager is to replace work done in spreadsheets. This use-case offers a number of benefits. For example, it is in line with a recent technology feature in *Nature*, which argues that the concept of reproducibility extends to creating easy-to-update analysis pipelines ^10^. With Vectools, these easy-to-update pipelines will also be easy to share, making it a valuable tool for bioinformatics research.

## Data availability

All data used in the paper are archived in Zenodo ^7^.

## Software availability

**Source code available from:** https://vectools.bitbucket.io/.

**Data and analysis pipelines:** http://doi.org/10.5281/zenodo.1413666 ^7^.

**Source code at time of publication:** http://doi.org/10.5281/zenodo.1413671 ^6^.

**License:** The software, and data and analysis pipelines are available under a Creative Commons Attribution 4.0 International (CC BY 4.0) license.

## Supplementary material

**Supplementary File 1. A list of operations offered by Vectools with short descriptions of their functions**
