# Supplementary material for: Increasing workflow development speed and reproducibility with Vectools
Source: F1000Res. 2018 Oct 23;7:1499. Originally published 2018 Sep 20. [Version 2] doi: 10.12688/f1000research.16301.2 (PMC6281013; doi:10.12688/f1000research.16301.2)
Supplement: Supplementary file 1 [file f1000research-7-18308-s0000.tgz › 3ca4ea15-8efd-4d60-8a85-ae51d714d7de_Supplementary_File_1.docx]

**Operations available in Vectools**

Analysis and Statistics

confmat - Generates a confusion matrix from a set of predictions.

divide - Divides values in a table by another.

max - Returns a vector containing the maximum value for each column in a matrix.

mean - Returns a vector containing the mean/average value for each column in a matrix.

median - Returns a vector containing the median value for each column in a matrix.

min - Returns a vector containing the minimum value for each column in a matrix.

mode - Returns a vector containing the mode for each column in a matrix.

pca - Maps data to a lower dimensional space with using principal component analysis.

pearson - Calculates the Pearson correlation coefficient with a cutoff at a given threshold.

percentile - Returns a vector containing the percentile for each column in a vector.

roc - Calculates the receiver operating characteristic (ROC) curve for a binary classification.

sd - Returns a vector containing the standard deviation of values within each column in a matrix.

shape - Finds the shape of a matrix.

spearman - Calculates the Spearman's rank correlation coefficient.

Descriptors

ncomp - Calculates the n-composition of a FASTA sequence (default: amino acid composition).

summary - Summarizes a matrix in a column-wise manner.

trans - Calculates the percent transitions of a single character or group to another.

Manipulation

aggregate - Aggregate svalues into rows based on key-columns.

append - Appensd values to a given matrix row or column wise.

chop - Removes rows from a matrix.

colmerge - Combine or split columns using a delimiter.

concat - Concatenates two matrices at a given axis.

creatematrix- Creates matrices filled with a value or special matrices, such as identity matrices.

format - Converts between various vector formats.

join - Joins two or more matrices on one or more columns.

slice - Manipulates matrix columns.

sort - Sorts a vector based on columns given by keys.

transpose - Transposes a matrix.

unique - Returns unique rows in a matrix.

vrep - Returns rows which contain a given set of elements.

Math

add - Adds matrices or scalars to a matrix.

determinant - Calculates the determinant for square matrices.

dotproduct - Calculates the dot product of two vectors.

eigenvalues - Calculates the eigenvalues of a matrix. The order is the same as in the function eigenvectors.

eigenvec - Calculates the eigenvectors of a matrix. The order is the same as the function eigenvalues.

inverse - Calculates the inverse matrix for square matrices.

multiply - Multiplies matrices via matrix-multiplication or scalars.

subtract - Subtracts matrices or scalars from a matrix.

sum - Sums the columns of a matrix.

Normalization

medpolish - Normalizes a matrix via median polish normalization.

quantnorm - Normalizes a matrix via quantile normalization normalization.

zscorenorm - Normalizes a matrix via z-score normalization normalization.

Supervised Learning

linreg - Performs linear regression via least squares on a set of vectors.

svmclassify - Predicts the class of unknown vectors using an SVM model.

svmtrain - Performs k-fold testing followed by independent set testing on a set of training vectors.

Unsupervised Learning

affcl - Performs affinity clustering on a set of vectors.

dbscan - Performs density based clustering of a set of vectors.

hierarc - Performs hierarchical clustering and returns cluster assignments or a linkage matrix.

kmeans - Performs k-means clustering on a set of vectors.

silscore - Calculates the silhouette score of a set of clusters.
