# Supplementary material for: Increasing workflow development speed and reproducibility with Vectools
Source: F1000Res. 2018 Oct 23;7:1499. Originally published 2018 Sep 20. [Version 2] doi: 10.12688/f1000research.16301.2 (PMC6281013; doi:10.12688/f1000research.16301.2)
Supplement: Peer review history

# Peer review: Referee response for version 2

**DOI:** 10.5256/f1000research.18308.r40346

**Published:** 2018-12-03

**Authors:** Dimitar Vassilev

**Affiliations:** 1 Faculty of Mathematics and Informatics, Sofia University “St. Kliment Ohridski”, Sofia, Bulgaria

The submitted material concerns a useful domain in applicative bioinformatics - development of pipelines and platforms for more or less general purposes and services in bioinformatics analyses. The major objective is to suspend the reliance of custom designed scripts and to improve the reproducibility of such software solutions.

 I think that the authors have managed to a large extent the improvement of the reproducibility of their software’s set of tools for vector and table manipulation, including also some basic statistical inferences and machine learning models.

 The efforts of the authors to have a formal general purpose platform at the end promised to have an obvious potential. Although, it is bold to speak about the certain large scale generality of the presented software – Vectools - because of the limited number of models incorporated.

 The lack of some very new models in Vectools is an open question and I hope that the authors are able to support the openness of the software and its future improvement. In regards to the performance speed of Vectools - it will be good to include a sort of api concerning some valuable information for speed and memory usage (which is not an easily-predicted problem in machine learning models).

 In regards to whether sufficient details of the code, methods and analysis were provided to allow replication of the software development and its use by others, I have answered ‘Partly’ to this question, because I think it would be worth providing the users of the software product with better documentation, which does not diminish the positive sides of the submitted material and the code.

 In regards to whether sufficient information is provided to allow interpretation of the expected output datasets and any results generated using the tool, the interpretation of the expected outputs is specific to every case, and depends very much on the data so should not be always certain in the very proper discussion of the results. In this line I would advise the authors to show a couple of references with wider theoretical basis for better discussion and interpretation of the outcomes of the submitted software product. 

 In regards to whether the conclusions about the tool and its performance are adequately supported by the findings presented in the article, I think the conclusions serve to the submitted material very well but it would be worth showing the specificity of the software product in order to avoid the implied total generality of the work.

 Finally, I support the submitted material to be indexed, with some amendments.

I have read this submission. I believe that I have an appropriate level of expertise to confirm that it is of an acceptable scientific standard.

# Peer review: Referee response for version 2

**DOI:** 10.5256/f1000research.18308.r39811

**Published:** 2018-10-29

**Authors:** Yutaka Saito

**Affiliations:** 1 Artificial Intelligence Research Center, National Institute of Advanced Industrial Science and Technology (AIST), Tokyo, Japan

In this revision, the authors have addressed all of my comments.

 I think the article is now suitable for indexing.

I have read this submission. I believe that I have an appropriate level of expertise to confirm that it is of an acceptable scientific standard.

# Peer review: Referee response for version 1

**DOI:** 10.5256/f1000research.17808.r38645

**Published:** 2018-09-26

**Authors:** Yutaka Saito

**Affiliations:** 1 Artificial Intelligence Research Center, National Institute of Advanced Industrial Science and Technology (AIST), Tokyo, Japan

This article describes Vectools, a command-line tool that can do various kinds of matrix operations for tsv-like data with simple one-liner programs. Vectools is similar to sed and awk commands in Unix Coreutils but has more functionalities, thereby reducing the cost for implementing custom scripts for daily data analyses. The authors claim this will improve the reproducibility problem in recent bioinformatics studies.

 As a general comment, I think Vectools is useful and will be of interest for bioinformaticians who work in practical data analyses. Although I do not feel the tool has a theoretical novelty, its practical usefulness is worth post-publication evaluation by future users.

 I have several comments as follows:

 1:

- Vectools is also similar to "groupby" function in Bedtools.  
- Some functionalities of Bedtools groupby are not included in Vectools, and vice versa.
- The authors should refer to Bedtools, and if any, other command-line tools similar to Vectools.

 

 2:

- For each analysis in Figure 1, please provide the size of input data (#rows, #columns, #sequences, etc.).
- Especially, I get the impression that SVM consumes a large memory.
- Although I partly agree with the authors' statement that the computational cost is a minor issue, it is still important to provide the information of memory usage along with data size.

3 (minor points):

- (Top left in page 2) However --> In addition (?)
- (Top right in page 3) valuable tool --> valuable tools

I have read this submission. I believe that I have an appropriate level of expertise to confirm that it is of an acceptable scientific standard, however I have significant reservations, as outlined above.

## References

[1] QuinlanARHallIM: BEDTools: a flexible suite of utilities for comparing genomic features. *Bioinformatics*.2010;26(6) : 10.1093/bioinformatics/btq033 841-2 10.1093/bioinformatics/btq033 20110278PMC2832824

# Response

**Published:** 2018-10-19

**Authors:** Shizuka Uchida

**We would like to thank the reviewer for valuable comments. The followings are our point-by-point responses:**

**> Comment #1: I have several comments as follows:**

- **Vectools is also similar to "groupby" function in Bedtools.  **
- **Some functionalities of Bedtools groupby are not included in Vectools, and vice versa.**
- **The authors should refer to Bedtools, and if any, other command-line tools similar to Vectools.**

*> Our response: Thank you very much for raising this point. We now clearly cite Bedtools in the Discussion section. To address the functionality issue, we have implemented two additional operations in Vectools, which are: 1) “mode” for calculating mode/antimode in Vectools; and 2) “colmerge” for combining or splitting columns based on a delimiter. We have also added the “--group” option to relevant operations (e.g., mean, mode, stdev). For cases in which the operation names or functionality do not match exactly, we list the equivalences between Bedtools Groupby and Vectools below:*

 Groupby - Vectools 

 count– shape | slice

 collapse– aggregate

 distinct– unique 

 count_distinct– unique | sum

 sstdev– vrep | stdev 

 freqasc/ freqdesc– unique | slice | colmerge | aggregate

 first/ last– chop

**> Comment #2:**

- **For each analysis in Figure 1, please provide the size of input data (#rows, #columns, #sequences, etc.).**
- **Especially, I get the impression that SVM consumes a large memory.**
- **Although I partly agree with the authors' statement that the computational cost is a minor issue, it is still important to provide the information of memory usage along with data size.**

*> Our response:* *We have updated the figure by adding the file sizes for the SVM example. The first two examples display the entire file. Thus, we did not add file sizes in those examples. We have updated the figure legend to make this clearer. Further, all data used is assessable in the archived data. We have also updated the figure legend to make this more apparent. Finally, we fixed two typos in the figure.*

**> Comment #3 (minor points): **

- **(Top left in page 2) However --> In addition (?)**
- **(Top right in page 3) valuable tool --> valuable tools**

*> Our response: Thank you very much for reading our manuscript carefully. We have corrected the above grammatical errors as well as others.*
